# New insights in gene expression alteration as effect of doxorubicin drug resistance in triple negative breast cancer cells

**DOI:** 10.1186/s13046-020-01736-2

**Published:** 2020-11-13

**Authors:** Cristina Alexandra Ciocan-Cartita, Ancuta Jurj, Oana Zanoaga, Roxana Cojocneanu, Laura-Ancuta Pop, Alin Moldovan, Cristian Moldovan, Alina Andreea Zimta, Lajos Raduly, Cecilia Pop-Bica, Mihail Buse, Liviuta Budisan, Piroska Virag, Alexandru Irimie, Sandra Martha Gomez Diaz, Ioana Berindan-Neagoe, Cornelia Braicu

**Affiliations:** 1 Research Center for Functional Genomics, Biomedicine and Translational Medicine, “Iuliu Hatieganu” University of Medicine and Pharmacy, Cluj-Napoca, Romania; 2 MedFuture Research Center for Advanced Medicine, “Iuliu Hatieganu” University of Medicine and Pharmacy, Cluj-Napoca, Romania; 3 Laboratory of Radiotherapy, Radiobiology and Tumor Biology, “Prof. Dr. Ion Chiricuta” Oncology Institute, Cluj-Napoca, Romania; 4 Department of Surgical Oncology and Gynecological Oncology, “Iuliu Hatieganu” University of Medicine and Pharmacy, Cluj-Napoca, Romania; 5 Department of Surgery, “Prof. Dr. Ion Chiricuta” Oncology Institute, Cluj-Napoca, Romania; 6 Brazilian Biosciences National Laboratory (LNBio), Brazilian Center for Research in Energy and Materials (CNPEM), Campinas, Sao Paulo 13083-970 Brazil; 7 Department of Functional Genomics and Experimental Pathology, “Prof. Dr. Ion Chiricuta” Oncology Institute, Cluj-Napoca, Romania

**Keywords:** Triple negative breast cancer, Doxorubicin, Next-generation sequencing, Microarray, Drug resistance

## Abstract

**Background:**

Triple negative breast cancer (TNBC) is a heterogeneous disease with aggressive behavior and an unfavorable prognosis rate. Due to the lack of surface receptors, TNBC must be intensely investigated in order to establish a suitable treatment for patients with this pathology. Chemoresistance is an important reason for therapeutic failure in TNBC.

**Method:**

The aim of this study was to investigate the effect of doxorubicin in TNBC cell lines and to highlight cellular and molecular alterations after a long exposure to doxorubicin.

**Results:**

The results revealed that doxorubicin significantly increased the half maximal inhibitory concentration (IC_50_) values at P12 and P24 compared to parenteral cells P0. Modifications in gene expression were investigated through microarray technique, and for detection of mutational pattern was used Next Generation Sequencing (NGS). 196 upregulated and 115 downregulated genes were observed as effect of multiple dose exposure, and 15 overexpressed genes were found to be involved in drug resistance. Also, the presence of some additional mutations in both cell lines was observed.

**Conclusion:**

The outcomes of this research may provide novel biomarkers for drug resistance in TNBC. Also, this activity can highlight the potential mechanisms associated with drug resistance, as well as the potential therapies to counteract these mechanisms.

**Supplementary Information:**

**Supplementary information** accompanies this paper at 10.1186/s13046-020-01736-2.

## Background

Triple-negative breast cancer (TNBC) is immunohistochemically defined by the lack of three important receptors: estrogen (ER), progesterone (PR) and human epidermal growth factor receptor 2 (HER2) [1, 2]. TNBC is a less frequent phenotype, being around 15–20% of all breast cancers. Despite this, TNBC is generally diagnosed in young patients, presenting a high metastatic capacity and an unfavorable prognostic rate [3]. TNBC is a relevant clinical challenge considering that this cancer subtype does not respond to endocrine therapy or other targeted agents [1, 4]. Meanwhile, conventional chemotherapy and radiotherapy remain the main important alternative for these patients [5].

Chemotherapeutics is widely used as treatment strategy against TNBC tumors. However, the effectiveness of the treatment can be affected by the activation of the resistance related mechanisms [5, 6]. A typical and common treatment used for breast cancer, is represented by doxorubicin (DNA damaging agent) in combination with paclitaxel (microtubule-stabilizing drug) or/and cyclophosphamide [7–9].

The mechanisms of primary or acquired chemoresistance to doxorubicin still remain to be deciphered. Thus, our study is focused on the regulatory pathways responsible for resistance to therapy and possible specific targets that could help optimize patient responses to this drug [10, 11]. Chemoresistance is correlated with genetic alterations, that can activate pro-survival signaling, DNA damage repair, drug efflux or epithelial-mesenchymal transition [12–14]. The recognition of molecular features responsible for a particular response to chemotherapy, particularly transcriptomics and genetic alterations, proved to have a significant impact on cancer research. These features could be represented by biomarkers for resistance or sensitivity to a particular drug, or specific mechanistic alteration that can be a starting point for overcoming this resistance mechanism [15].

In this study, we developed two doxorubicin-resistant TNBC cell lines (Hs578T/Dox and MDA-MB-231/Dox) by multiple dose exposure of TNBC cells to doxorubicin, followed by evaluation of the alteration at genetic and transcriptomic levels. In this sense, the evaluation of gene expression patterns as effect of multiple dose exposure to doxorubicin was explored. The response of TNBC cell lines (Hs578T and MDA-MB-231) to doxorubicin was examined after exposure to 50 nM doxorubicin for 12, respectively 24 passages, followed by the evaluation of morphological alterations, along with genetic and transcriptomic patterns.

## Materials and methods

### Cell culture and induction of doxorubicin resistance

In this study, the experiments were performed on triple negative breast cancer (TNBC) cell lines, Hs578T and MDA-MB-231. The Hs578T cell line was cultured in D-MEM high glucose (D-MEM Gibco®) supplemented with 10% fetal bovine serum (FBS- Gibco®), 2 mM L-glutamine (Gibco®), 1% MEM Non-Essential Amino Acids Solution (100X, Gibco®), 0.01 mg/ml insulin and 1% Penicillin-Streptomycin (Gibco®). MDA-MB-231 cell line was cultured in RPMI-1640 (RPMI-1640 Gibco®), supplemented with 10% fetal bovine serum (FBS- Gibco®), 2 mM L-glutamine (Gibco®) and 1% Penicillin-Streptomycin (Gibco®). Cells were maintained in a humidified atmosphere at 37 °C with 95% air and 5% of CO_2_ (carbon dioxide). The doxorubicin-resistant TNBC cells were established by multiple dose exposure. The drug concentration used for maintaining the drug resistance of Hs578T/Dox and MDA-MB-231/Dox was 50 nM. Cells were treated with this dose for 12, and respectively 24 passages (Figure S1).

### Assessment of doxorubicin sensitivity

The assessment of the half maximal inhibitory concentration (IC_50_) of the parental (Hs578T and MDA-MB-231) and drug-resistant cells (Hs578T/Dox and MDA-MB-231/Dox) was performed through MTT assays in order to evaluate the inhibitory effects on cell proliferation. In brief, at a seeding density of 1.2 × 10^4^ cells/well, the cells were plated in 96-well plates and treated with stepwise concentrations of doxorubicin. Cellular viability and cytotoxicity were evaluated after 48 h by adding 1 mg/ml MTT solution and withdrawn after 2 h of incubation. As a final step, 100 μl of dimethyl sulfoxide was added in each well and the absorbance was detected at 570 nm using a BioTek microplate reader.

### Cytoskeletal evaluation

Morphological traits were evaluated through confocal microscopy using specific dyes for actin-filaments (Phalloidin, green dye) and nucleus (DAPI, blue dye). Modifications that occur post-therapy were evaluated at passage P0, P12 and P24 in order to increase the effects of doxorubicin on both TNBC cell lines. For this evaluation, treated cells were fixed and permeabilized with 4% paraformaldehyde, respectively 0.5% Triton X. Moreover, treated cells were stained with 200 nM Phalloidin dye for 30 min followed by an additional 1 min incubation with DAPI dye. The coverslips were mounted with 90% glycerol. The fluorescence microscopically images were captured using UPLSAPO40X2 (NA:0.95, Olympus Japan).

### DNA fragmentation using comet assay

DNA fragmentation using Comet assay as effect of serial exposure to doxorubine (P0, P12 and P24) on Hs578T and MDA-MB-231 was evaluated using the alkaline single cell gel electrophoresis assay or Comet assay, by Tice’ protocol as described previously [16, 17].

### Genetic alteration evaluation using next-generation sequencing panel on ion torrent

DNA was extracted using the Purelink Genomic DNA minikit following the manufacturer instruction. We used two triple negative breast cancer cell lines (HS578T and MDA-MB-231) samples that were treated with doxorubicin. DNA was extracted from samples at passage 0 (P0), 12 (P12) and 24(P24) after treatment. The DNA concentration was quantified using NanoDrop and were obtained concentrations between 54.19–115.4 ng/μl.

20 ng of DNA were used for sequencing using the Ion AmpliSeq Cancer Hotspot Panel v2 (ThermoFisher Scientific) and the Ion AmpliSeq Library 2.0 kit (ThermoFisher Scientific). The Ion AmpliSeq Cancer Hotspot Panel v2 consists of primers for hotspot evaluation in the following genes: *ABL1, AKT1,ALK, APC, ATM, BRAF, CDH1, CDK2A, CSF1R, CTNNB1, EGFR, ERBB2, ERBB4, EZH2, FBXW7, FGFR1, FGFR2, FGFR3, FLT3, GNA11, GNAQ, GNAS, HNF1A, IDH1, IDH2, JAK2, JAK3, KDR, KIT, KRAS, MET, MLH1, MPL, NOTCH1, NPM1, NRAS, PDGFRA, PIK3CA, PTEN, PTPN11, RB1, RET, SMAD4, SMARB1, SMO, STK11, TP53, VHL*. After library preparation, the samples were purified using the AMpure XP Beads (Bechman Coulter). The purified libraries were quantified using the fluorometer Qubit 2.0 and the Qubit HS DNA kit. For template synthesis, libraries were diluted to 100pM and multiplex for libraries on Ion 316 Chip (ThermoFisher Scientific). The sequencing process was performed on the Ion Torrent PGM Machine (ThermoFisher Scientific) using the Ion PGM HI-Q Sequencing 200 kit. The data obtained after sequencing were analyzed using the Torrent Suit 5.6 and Ion Reporter 5.6 software for data trimming, alignment and variant calling. The obtained variants were filtered using the following conditions: *p* value ≤0.05, coverage ≥500.

### Gene expression microarray evaluation

Total RNA extraction, from TNBC treated and untreated cells, was performed using TriReagent (Invitrogen) and purified using RNeasy miniprep kit (Qiagen) according to the manufacturer’s instruction. The RNA concentration and quality were evaluated using Nanodrop-1000 spectrophotometer (Thermo Scientific) and Bioanalyzer (RIN ≥ 7).

The alteration of gene expression pattern was done using Agilent microarray technology using SurePrint G3 Gene Expression Microarrays (8x60k), covering 26,083 genes and 30,606 lncRNA transcripts starting from 200 ng of total RNA following the Agilent standard protocol. After hybridization step, 17 h at 65 °C at 10 rpm, the arrays were washed and scanned with the Agilent scanner. Probe features were extracted from the microarray scan data using Feature Extraction software (Agilent Technologies).

### qRT-PCR data validation

Validation of the microarray results was done using RT-PCR technique on both TNBC cells. In this regard, genes involved in drug resistance mechanisms were selected (IL-6, CLU, JUNB and TNSF10). GAPDH and B2M were used as reference genes. In brief, 1000 ng of total RNA was reversed transcribed into cDNA using High Capacity cDNA Reverse Transcription Kit (Applied Biosystems) and amplified using SYBR Select Master Mix (Applied Biosystems) on ViiA™7 System (10 μl reaction volume in 384-well plate). Relative quantification was done using the 2^-ΔΔCT^ method.

### CXCL1, IL-6 and TNF-α quantification in cell culture medium

The expression levels of CXCL1 released in the cell culture medium were detected by ELISA using the Human CXCL1 DuoSet ELISA (R&D System, cat no. **DY275**). For TNF-α was used Human TNF-α DuoSet ELISA (R&D System, cat no. **D210**), and IL-6 DuoSet ELISA (R&D System, cat no. DY206) for IL-6 quantification along with DuoSet Ancillary Reagent Kit 2 (R&D Systems, cat no. DY008).

### Statistical analysis

Resulted data were expressed as mean ± SD (standard deviation). The difference between experimental conditions and controls were analyzed using t test (statistically significant was considered *p* < 0.05). Statistical analyses were carried out using GraphPad Prism version 6, Panther, Venny and String 8.0 free version software.

## Results

### Cellular viability and doxorubicin assessment on TNBC cell lines

**Fig. 1:**
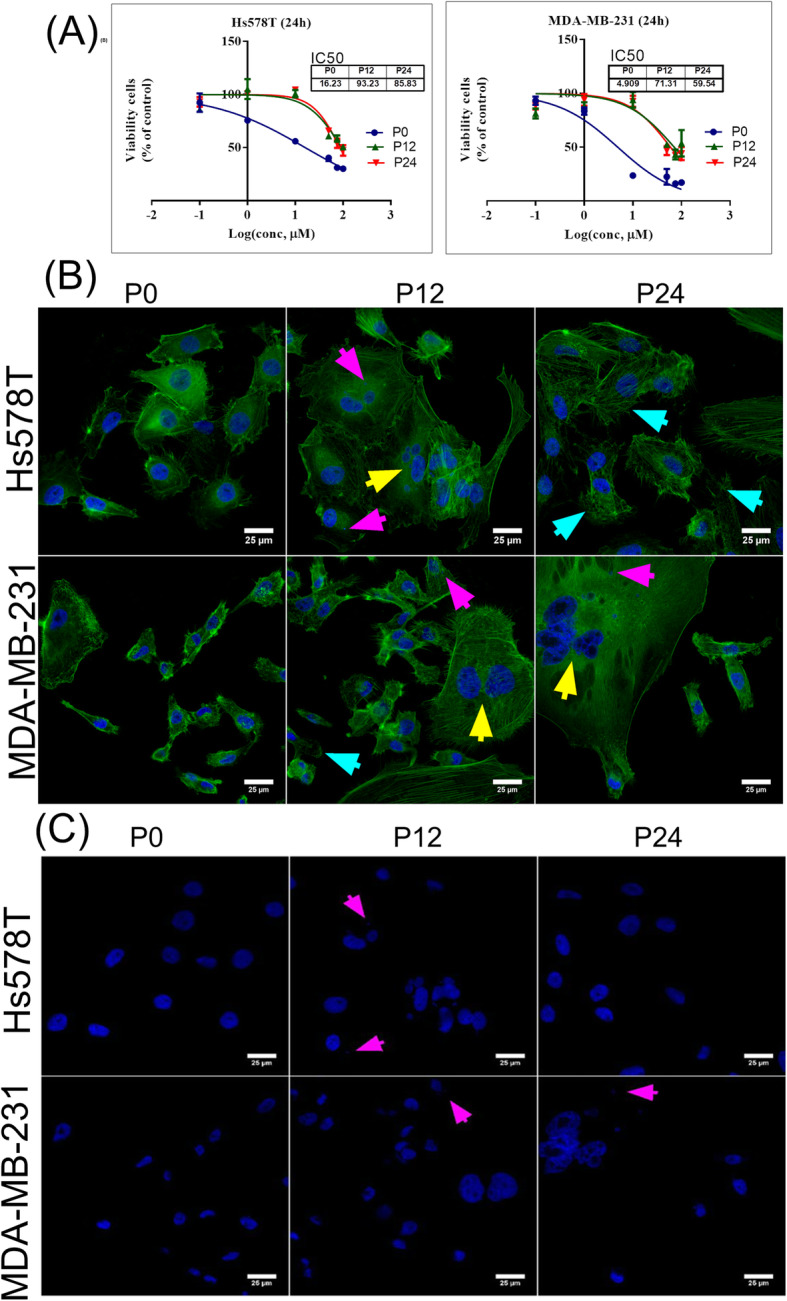
The effects on cell proliferation and cell morphology after doxorubicin-multiple dose exposure. **a** The antiproliferative effects on TNBC cell lines, Hs578T and MDA-MB-231, measured using MTT assay using stepwise concentrations of doxorubicin (0.1, 1, 10, 50, 75, 100 μM) to determine the IC_50_ values at P0, P12 and P24; data are represented as Log (conc, μM) = log[concentration of doxorubicin, μM] (mean ± SD, *n* = 3). **b** The evaluation of morphological traits after doxorubicin exposure on TNBC cell lines (P12 and P24) though confocal microscopy. Actin filaments were staining using Phalloidin dye and nucleus was staining using DAPI dye. Scale represents 25 μm. Images were captured with 60x oil immersed objective (PLAPON60xOSC2,1.4 NA). **c** Alteration of nuclear cell morphology, evaluated by DAPI staining on blue channel (405 nm excitation). Magenta arrows represent the formation of the micronucleus, yellow arrows point multinucleate/polynuclear cells and blue arrows point cytoskeleton damage

The effect of doxorubicin on TNBC cell lines was investigated in control cells (P0), cells with multiple dose exposure for 12 serial passages (P12), respectively 24 passages (P24), with doxorubicin between 0 and 100 μM. The MTT values are presented as % of control in relation with the log (concentration, μM) (Fig. 1a). IC_50_ concentrations were calculated at each time point using GraphPad Prism software. According to IC_50_ concentrations, important modifications in antiproliferative activity of treated TNBC cells were observed. The increased values of IC_50_ corresponding for passage P12 respectively P24 are related to the activation of drug resistance mechanisms.

Further, morphological traits were investigated as a result of a long-term exposure to doxorubicin therapy. In Fig. 1b the significant alterations occurred in cellular morphology are highlighted through the presence of modifications in actin-filaments and nucleus structure. In the control group, both cell lines exhibited normal features, meanwhile treated TNBC cells presented modifications in their structure. After doxorubicin exposure both cells lines started to exhibit alterations in their structure suggesting the activation of apoptotic processes, as compared to the control cells (P0). Thereby, the presence of micronucleus (magenta arrows), cytoskeleton damage (blue arrow) and polynuclear cells (yellow arrow) were showed at P12 and 24 in Hs578T and MDA-MB-231 cells, respectively after a prolonged exposure with doxorubicin. No such alterations were observed in P0 cells. The micronucleus alteration is detailed in Fig. 1c, by nuclear DAPI staining, on blue channel. Also, MDA-MB-231 cells treated at passage P12 and P24 exhibited giant cells suggesting that doxorubicin induces cell death through mitotic catastrophe where cells become multinucleated and enlarged, as well as loss of plasma membrane integrity. The alterations observed in Hs578T P24 were associated with stress fibers that are related to the EMT process and to the mesenchymal traits, proving that doxorubicin treatment activates more aggressive cells.

### DNA fragmentation using comet assay

**Fig. 2:**
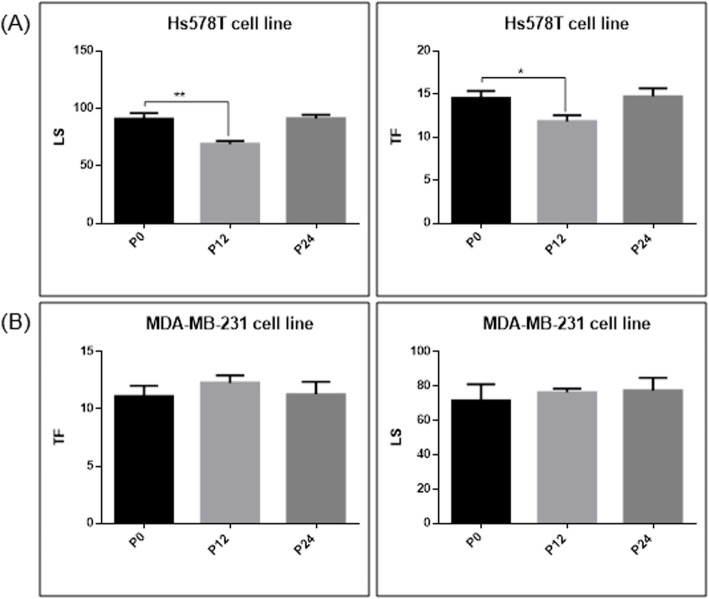
LS and TS calculated for the treatment with doxorubicine serial passages (P0: control cells, P12 and P24 **a** Hs578T cells and **b** MDA-MB-231 cells

DNA breaks (single or double strand breaks) leads to a relaxation of the DNA, migration under electric field creating comet tails. DNA cross-links do not permit DNA unwinding. Thus, when they are produced, the DNA does not migrate and comet tails are shorter. The treatment with doxorubicin led to DNA lesions which were observed under the microscope as comet tails of different grades, being described as lesion scores (LS) and tail factor (TF), as presented in Fig. 2.

DNA breaks form comet tails with different lengths depending on the severity of the lesions. In our experiment the exposure to doxorubicin did not produce significant difference between the LS and TF, compared to control cells (P0) in both cell lines, except for the Hs578T cells at P12, where the LS and TF values were lower than the control cells, probable as effect of DNA repair mechanism activation. This phenomenon was not retrieved at P24, meaning that DNA breaks were similar as in control P0 cells.

### Identification of mutation signatures in drug-resistant TNBC cells

**Fig. 3:**
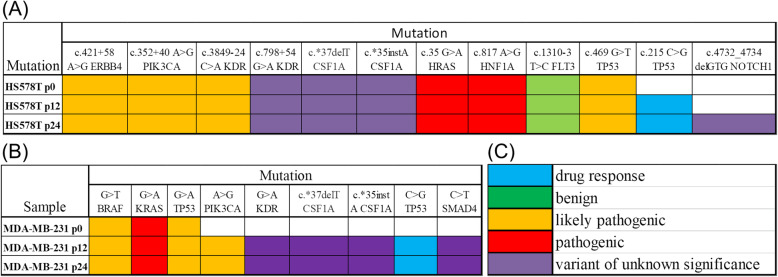
Mutation patterns in TNBC cells evaluated by next generation sequencing using Ion Torrent PGM Machine and Ion AmpliSeq Cancer Hotspot Panel. **a** Mutations identified in the HS578T samples; **b** Mutations identified in the MDA-MB-231 samples; **c** type of mutation legend: blue- drug response, green- benign, orange-likely pathogenic, red- pathogenic, purple- variance of unknown significance

We analyzed the mutation patterns for both TNBC cell lines, Hs578T and MDA-MB-231, at passage P0, P12 respectively P24. Figure 3a and b present the mutations identified in each gene for each passage. In the case of Hs578T cell line, we observed that all three passages present the same mutations signature, with two exceptions; mutation c.215C > G in *TP53* gene presented in both passages, P12 respectively P24, as well as the presence of mutation c.4732_4734delGTG in *NOTCH1* gene presented in passage P24. Also, the mutation presented in *TP53* gene is associated with drug response in clinical database ClinVar (Fig. 3). Meanwhile, the mutation observed in *NOTCH1* gene exhibits unknown clinical implication (based on ClinVar or FATHMM data base) but is already described in the public databases dbSNP and COSMIC, the clinical significance of this mutation remains to be demonstrated. For MDA-MB-231 cell line, the mutation signatures are similar for passage P12 and P24. For passage P0 (used as control) we found only the presence of three mutated genes, *BRAF*, *KRAS*, *TP53*. The mutated genes in TNBC cell lines have both intronic and exonic localization. *TP53* has a very low activity in the analyzed cell lines. As can be observed in the IntoGene software, the main driver genes in breast cancer are *TP53* and *PIK3CA*. Also, by reevaluating the CliVar database we observed that mutations of *ERBB4* (c.421 + 58 A > G), *PIK3CA* (c.352 + 40 A > G) and *KDR* (c.3849–24C > A) have unknown significance (Fig. 3). Also, the *TP53* c.469C > T was observed in some studies on breast cancer and classified as likely pathogenic or pathogenic [18, 19]. The *TP53* c.839G > A mutation was also observed in early onset familial prostate cancer and classified as likely pathogenic [20].

### Identification of altered genes and lncRNAs expression profiles in TNBC cell lines as effect of doxorubicin therapy

In the present investigation of expression profile was used Agilent microarray technology (8x60k slides) to identify the altered transcriptomics pattern as response to multiple dose exposure to doxorubicin therapy. Thus, it was possible the identification of the most relevant altered genes in TNBC cell lines, as well as in passage P12, respectively P24, versus control cells (P0). A cut-off value for FC of ±2 and *p* ≤ 0.05 was selected to determine the modifications occurred in transcriptomic patterns.

Significant alteration on gene and lncRNAs-expression profiles for both cell lines were identified, indicating the presence of 2035 altered genes (966 overexpressed and 1069 downregulated) and 1441 lncRNAs (534 overexpressed and 907 downregulated) on Hs578T cells at P12 versus P0; 1071 differentially expressed genes (510 overexpressed and 561 downregulated) and 835 differentially expressed lncRNAs (412 overexpressed and 423 downregulated) on MDA-MB-231 cells were identified at P12 versus P0.

**Fig. 4:**
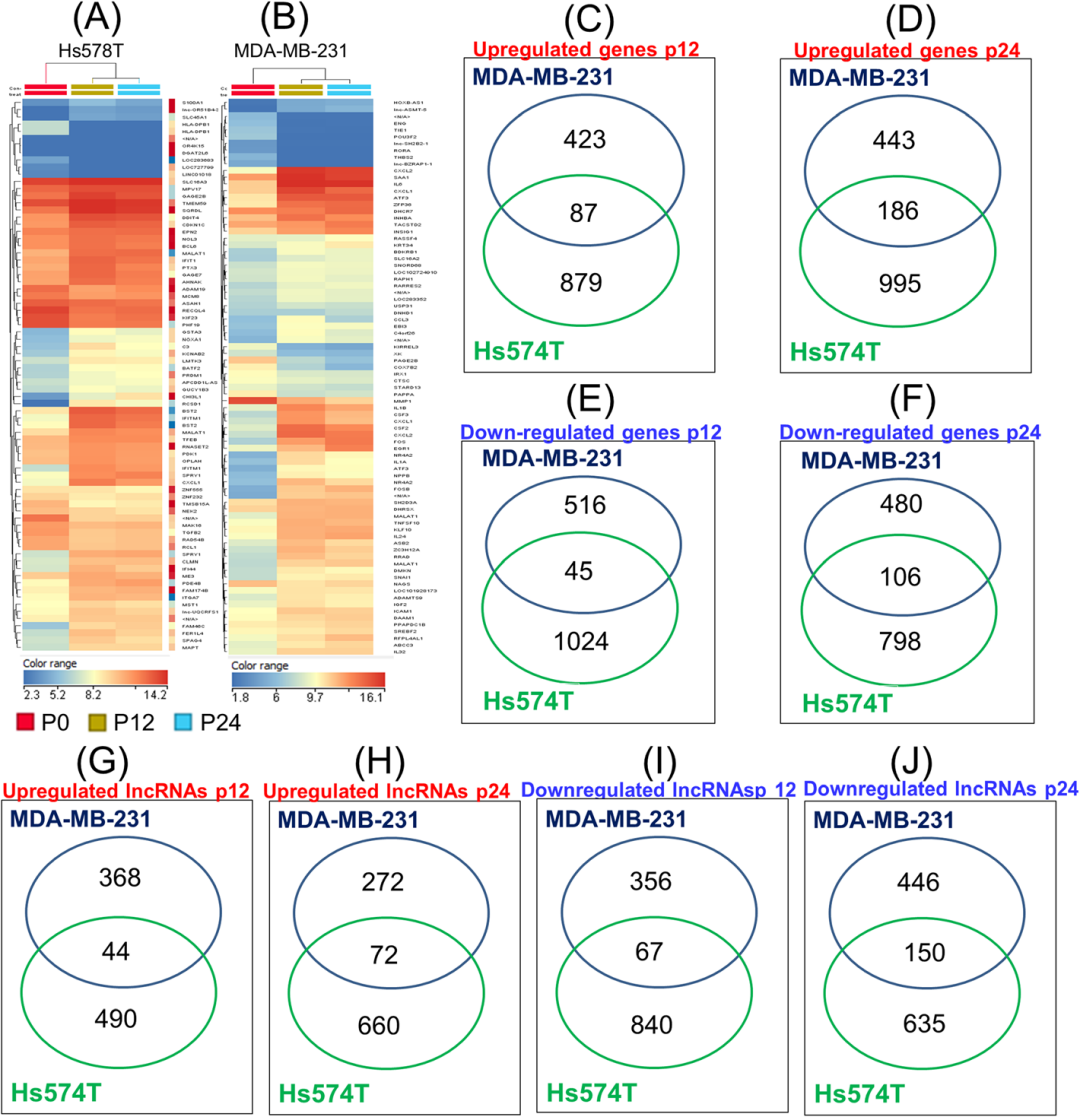
Gene expression profiling of TNBC cell lines as effect of multiple dose exposure on doxorubicin therapy. Genes Heatmaps emphasizing the altered genes signature at passage P12 and P24 in **a** Hs578T cell line and **b** MDA-MB-231 cell line. Venn diagram of the statistically determined (FC ± 2 and *p*-value ≤0.05) upregulated and downregulated genes expressions by overlapping both TNBC cell lines. **c**, **d** Highlighted the common overexpressed gene expression signature at P12, respectively P24; **e**, **f** common downregulated gene expression signature at P12, respectively P24; **g**, **h** common upregulated lncRNAs expression signature at P12, respectively P24; **i**, **j** common downregulated lncRNAs signature at P12, respectively P24

**Fig. 5:**
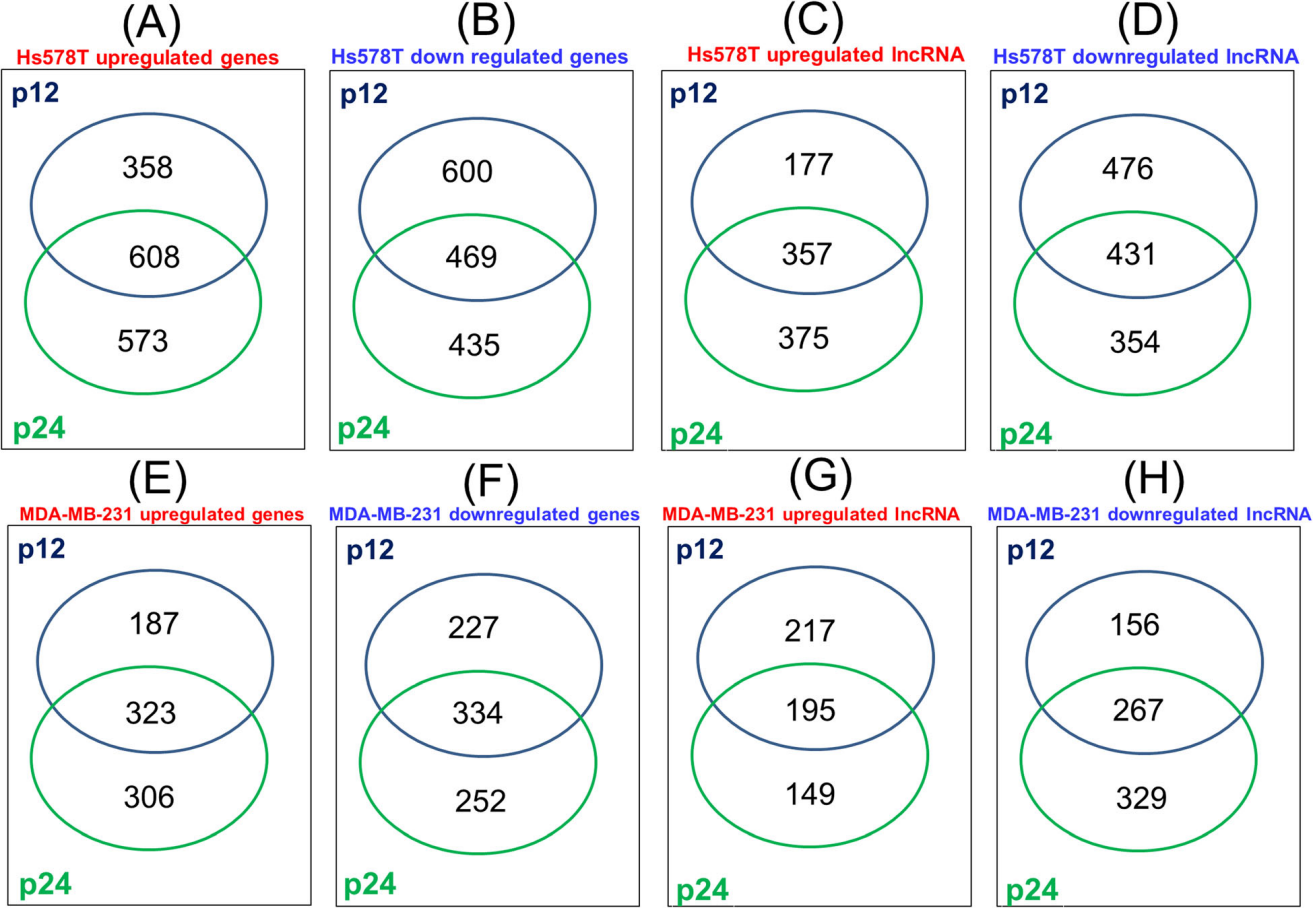
Venn diagram for altered coding genes and lncRNAs at p12 and p24 versus control cells (P0) on both TNBC cell lines **a** common overexpressed gene expression signature at p12, respectively p24 on Hs578T cell line. **b** downregulated gene expression signature at p12, respectively p24 on Hs578T cell line. **c** common overexpressed lncRNAs expression signature at p12, respectively p24 on Hs578T cell line. **d** downregulated lncRNAs expression signature at p12, respectively p24 on Hs578T cell line. **e** common overexpressed gene expression signature at p12, respectively p24 on MDA-MB-231 cell line. **f** downregulated gene expression signature at p12, respectively p24 on MDA-MB-231 cell line. **g** common overexpressed lncRNAs expression signature at p12, respectively p24 on MDA-MB-231 cell line. **h** downregulated lncRNAs expression signature at p12, respectively p24 on MDA-MB-231 cell line

Moreover, the expression profiles of 2085 altered genes (1181 overexpressed and 904 downregulated) and 1517 lncRNAs (732 overexpressed and 785 downregulated) on Hs578T cells P24 versus P0, as well as 1215 genes (629 overexpressed and 586 downregulated) and 940 lncRNAs (344 overexpressed and 596 downregulated) between MDA-MB-231 cells at P24 versus P0 were observed. The data are summarized in Tables 1 and 2, where top 20 altered transcripts on each experimental setting are presented. The heatmap for this data is presented in Fig. 4a and b. We overlapped the genes and lncRNAs profiling data obtained from the datasets mentioned above and the Venn diagram represented in Fig. 4c-j. The common genes and lncRNAs were altered in both TNBC cell lines in all three passages (P0, P12 respectively P24). In Fig. 5 are presented the common genes/lncRNAs in Hs578T and MBA-MB-231 cells. In this regard, we can observe the presence of a high number of genes/lncRNAs identical in both cell lines, transcripts involved mainly in modulation of different biological processes.

**Table 1:** Overview of the genes with an altered expression level as effect of exposure to 12 serial doses of doxorubicin (P12), respectively 24 serial doses (p24) versus control cells (P0), on Hs578T and MDA-MB-231, considering a cut-off value for FC ±2 and *p*-value ≤0.05

Cell line	Analysis	RNA species	Upregulated	Downregulated
Hs578T	P12 versus P0	Coding Genes	966	1069
		lncRNAs	534	907
	P24 versus P0	Coding Gene	1181	904
		lncRNAs	732	785
MDA-MB-231	P12 versus P0	Gene	510	561
		lncRNAs	412	423
	P24 versus P0	Gene	629	586
		lncRNAs	344	596

**Table 2:** Top 20 most abundant altered genes and lncRNAs as effect of exposure to 12 serial doses of doxorubicin (P12), respectively 24 serial doses (p24) on both TNBC cell lines, Hs578T and MDA-MB-231

Cell line	Analysis	RNA species	Upregulated	Downregulated
Hs578T	P12 versus P0	Gene	FOS, FOSB, PCSK2, RCSD1, CXCL2, EGR1, SLC12A7, CXCL2, CXCL3, KIR2DS4, IL6, PKNOX2, IFITM1, CXCL8, SLC14A1, MX1, IFI27, IFITM1, DRD1, OAS1	LCP1, HLA-DPB1, NCAM1, LPP, HAPLN2, WDR76, TRA2A, RHOJ, SH3GL3, ZBTB32, C3AR1, TAS2R30, CCDC177, PDE8B, FEM1A, CLDN10, KRT83, UTP11L, GALNT14, YIF1B
		lncRNA	lnc-ARRDC3–1, C8orf4, lnc-AC092327.1–3, LINC01082, LINC01016, LOC152286, LOC100506474, C1orf167, LOC100133669, LOC101928093, FER1L4, LOC100240735, FENDRR, MRVI1-AS1, XLOC_l2_012847, CA5B, LINC01431, LOC100506098, lnc-EPSTI1–3, SNORD114–2	TMEM200C, XLOC_l2_015885, RNA28S5, RNA18S5, lnc-CDKAL1–1, lnc-ANKRD53–1, TMPRSS4-AS1, lnc-C12orf49–2, SNRNP40, LOC441268, lnc-RASA1–3, LINC00271, LOC102031319, lnc-VPS4A-1, ZNF385D-AS2, lnc-TSC22D1–1, LOC100129940, lnc-CTBP1–1, HAAO, lnc-ANKRD11–2
	P24 versus P0	Gene	FOS, FOSB, PCSK2, IDO1, SLC12A7, ATF3, EGR1, CXCL2, SLC14A1, DRD1, ATF3, RCSD1, BMP7, NR4A2, COLEC12, GABRA2, ACKR3, NR4A3, CES1	LCP1, NAP1L3, NR0B1, NCAM1, HLA-DPB1, KRT83, FAM133A, NEFM, GALNT14, ST6GAL2, DOK5, MAGED4B, LMO7DN, SULT6B1, CILP, SH3GL3, EPB41L4A, SRD5A2, ITM2A, JPH1
		lncRNA	CYP2S1, lnc-ARRDC3–1, lnc-AC092327.1–3, C8orf4, LINC01082, lnc-GABRA2–1, LUCAT1, CYP2S1, LOC101928093, lnc-EPSTI1–3, lnc-KLHDC10–2, FENDRR, lnc-ENPP2–1, CA5B, XLOC_l2_013293, LINC00524, LOC100506098, LOC344887, FENDRR, LOC101928582	LOC100507377, SP3P, C16orf97, CAMK1D, LOC101928942, C1orf220, LINC01197, LOC101927115, lnc-CXCL2–1, lnc-MBP-1, LOC284009, lnc-AC078802.1–1, MCM3AP-AS1, lnc-RASA1–3, MKL2, lnc-PYDC2–1, lnc-ZNF91–2, HAAO, FLJ11710LOC100132495
MDA-MB-231	P12 versus P0	Gene	CXCL2, CCL20, CSF3, FOSB, NR4A3, NR4A2, FOS, CSF2, LCN2, IL6, NPPB, ATF3, HES1, IL1A, CXCL1, CXCL3, C3, ATF3, SAA2, IL1B	THBS2, HTR1F, ATP8A1, SSX4B, SSX1, BEX5, POU3F2, SNCAIP, TIE1, SEPP1, SHISA3, PCSK5, PPP2R2C, DMD, CHRDL1, KLHL10, PELI2, CHRM2, ATP11AUN, CMKLR1
		lncRNA	RNA5-8S5, LOC729451, C1QTNF1-AS1, lnc-MTHFD2L-1, C4orf26, LOC102724910, LOC101928353, MALAT1, lnc-FLYWCH2–1, LINC00996, lnc-FGF9–1, C15orf48, lnc-ASMT-5, LOC101926959, XLOC_l2_015438, SAT1, lnc-DZIP1–3, NAMPT, HOXB-AS1, lnc-IL6–2	lnc-IL1R2–1, lnc-POTEB-5, lnc-PRKD1–2, LOC643201, lnc-C14orf37–1, XLOC_l2_008221, SMEK3P, EPB41L4A-AS2, TMEM71, SSX8, XLOC_l2_012323, ZNF503-AS2, LOC101929484, lnc-RP1–177G6.2.1–2, TMEM92-AS1, lnc-GNLY-1, LOC101928710, LOC643201, lnc-MAFB-1, LOC101929395
	P24 versus P0	Gene	FOSB, FOS, CXCL2, CCL20, IL6, CSF3, ATF3, NR4A3, NR4A2, NPPB, CXCL3, NKX2–1, LCN2, CSF2, EGR1, EGF4, HES1, C1QTNF1, ZFP36, MUC4	THBS2, HTR1F, CHRDL1, ATP8A1, CMKLR1, SSX1, SSX4B, PCSK5, PAGE2B, PAGE2, POU3F2, PELI2, SEPP1, KIRREL3, COX7B2, TIE1, BEX5, KLHL10, GPR158, MMP1
		lncRNA	XLOC_l2_012748, lnc-MTHFD2L-1, LOC729451, lnc-ME3–1, MALAT1, lnc-ASMT-5, C11orf53, lnc-FGF9–1, LOC102724910, C1QTNF1-AS1, lnc-ANKRD10–1, LOC101926940, SNORD36B, HOXB-AS1, LOC100133669, lnc-UMPS-2, LOC102724434, XLOC_l2_013125, XLOC_l2_015438, C11orf96	lnc-POTEB-5, lnc-PRKD1–2, LOC101928942, XLOC_l2_012323, LOC643201, lnc-PTPRG-1, LINC01197, lnc-FOXL1–2, LOC101928915, LOC101928710, EPB41L4A-AS2, XLOC_l2_008783, LOC643201, SSX8, LOC100128242, LOC101928413, lnc-RP11-712 L6.5.1–2, lnc-BLID-1, lnc-CCDC140–5, lnc-JPH4–1

### Pathway analysis in TNBC cell lines

**Fig. 6:**
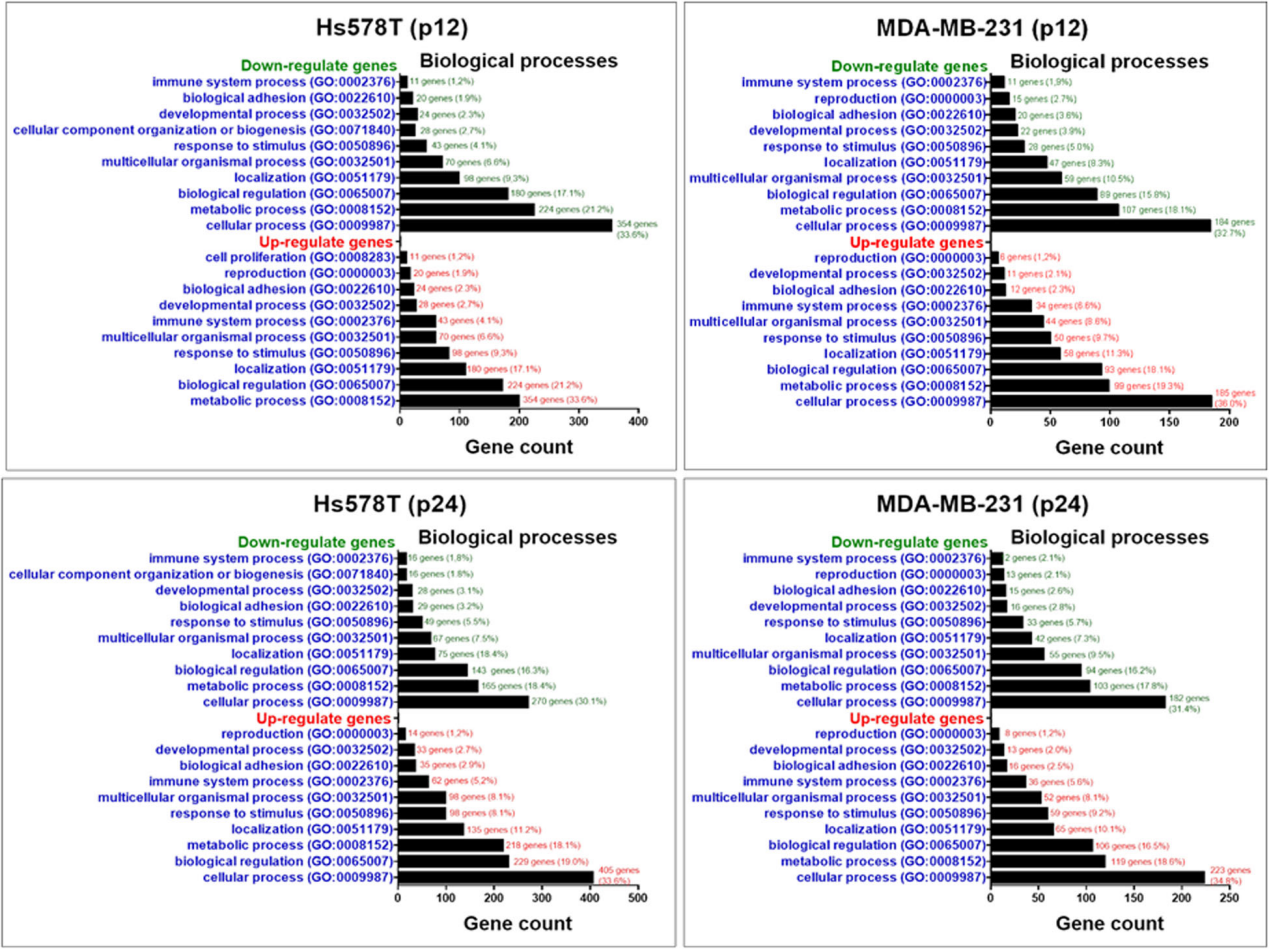
The main biological processes specific for up- and downregulated altered genes in TNBC cell lines at P12 and P24 using Panther software (http://www.pantherdb.org)

The genes with an altered expression level were input into specialized software in order to obtain the important biological processes activated after the exposure to doxorubicin. Panther software highlights the main altered biological functions based on the genes signature from the TNBC cell lines at passage P12, respectively P24. Therefore, 10 relevant biological processes were identified for P12 and P24 as can be observed in Fig. 6. Moreover, the biological processes between genes with down- or upregulated expression profile in P12 and P24 conditions are similar. The altered genes are mainly involved in cell proliferation and reproduction, and immune system processes.

**Fig. 7:**
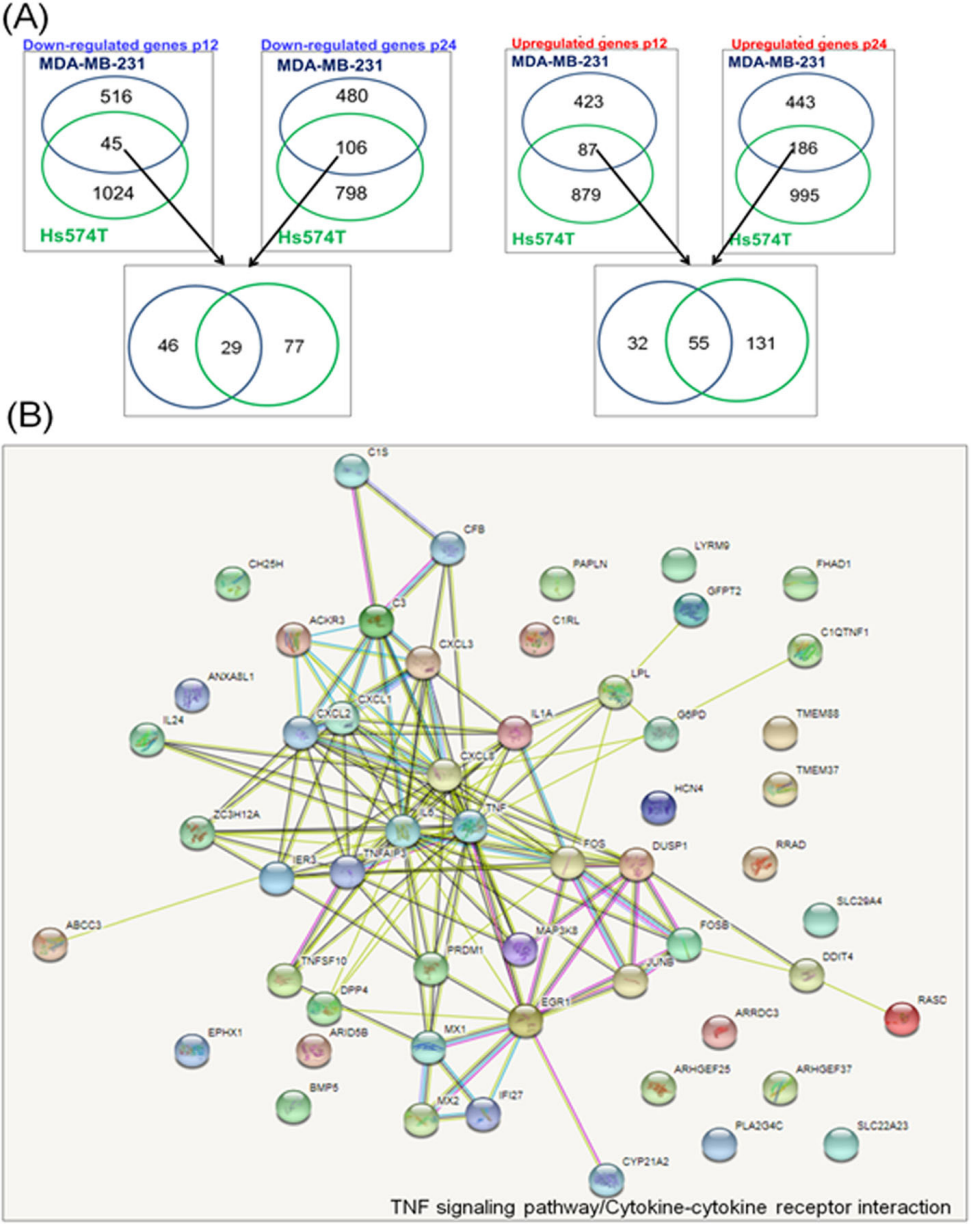
Common altered signature as effect of multiple dose exposure to doxorubicine **a** Common altered up- and downregulated genes between P12 and P24 in Hs578T and MDA-MB-231. **b** Pathway analysis of differentially expressed genes at P12 and P24 on Hs578T and MDA-MB-231 cell lines using String software

Using Venn diagram, the main common up- and downregulated genes between P12 and P24 in same cell lines were highlighted (Fig. 7a). The altered up- and downregulated genes were used to generate graphical representation of molecular networks using String software, which displays the specific interactions between transcripts (Fig. 7b). The altered genes critically regulated progression and cell fate by activation of tremendous processes such as TNF signaling pathway or cytokine-cytokine receptor interaction.

**Fig. 8:**
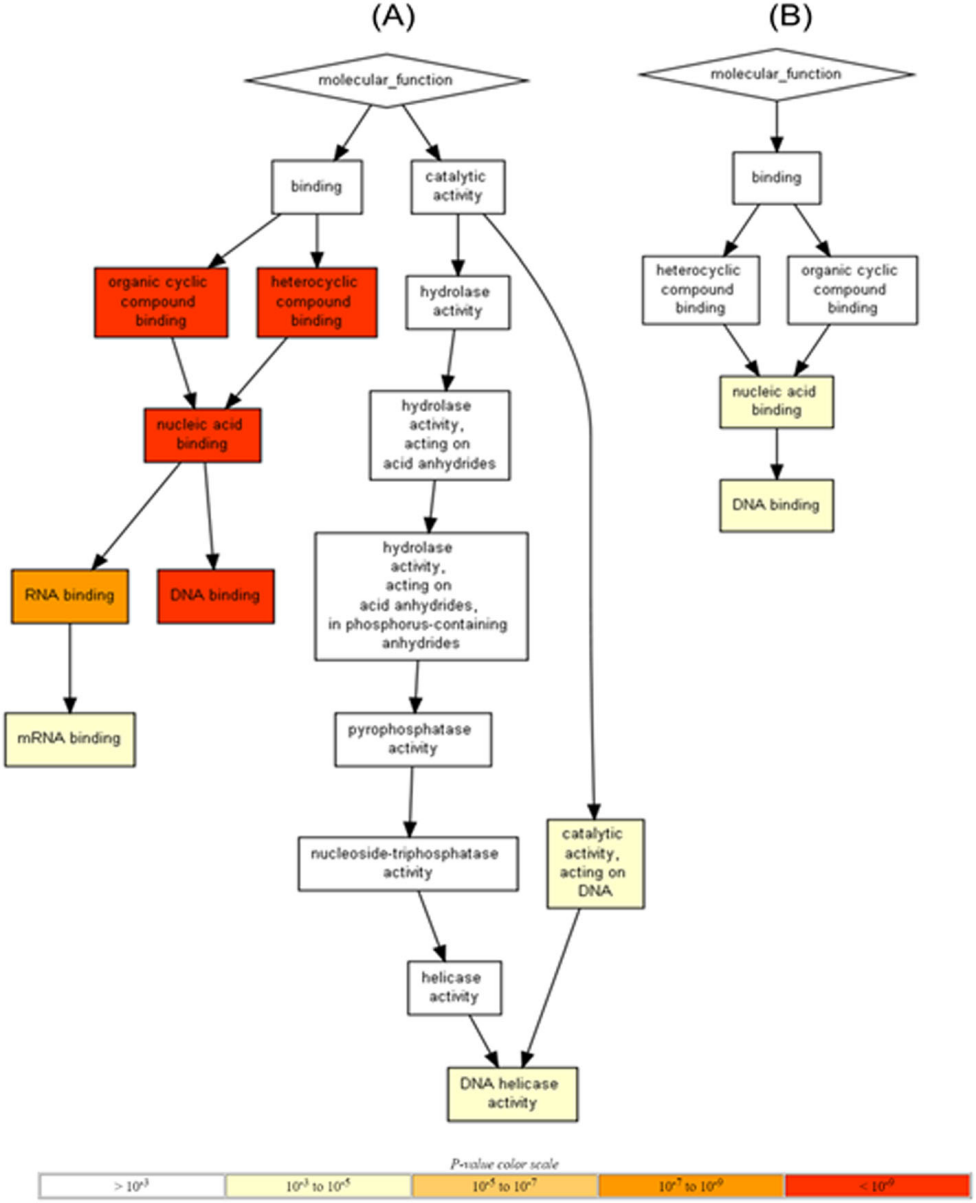
Gene enrichment analysis using GOrilla (Gene Ontology enrichment analysis and visualization tool) for **a** Hs578T and **b** MDA-MB-231 cell line

Altered genes can regulate essential processes responsible for cancer development. Therefore, an additional gene enrichment analysis using GOrilla software presents the involvement of this alterations in different molecular functions proving that doxorubicin has intricate features on nucleic acid binding activity and particular DNA binding. This gene enrichment analysis highlighted the fact that Hs578T cell line (Fig. 8a) exhibits more altered processes compared to MDA-MB-231 cell line (Fig. 8b).

### Construction of a gene expression network involved in drug resistance

Despite important progress in cancer treatment, acquired resistance to chemotherapeutic drugs still remain a major obstacle in patient treatment and overall outcome. Anticancer drug resistance is activated via numerous mechanisms, and microarrays technique offer a new approach to analyze cellular pathways involved in drug resistance mechanisms being used to predict the unexpected side effects.

**Fig. 9:**
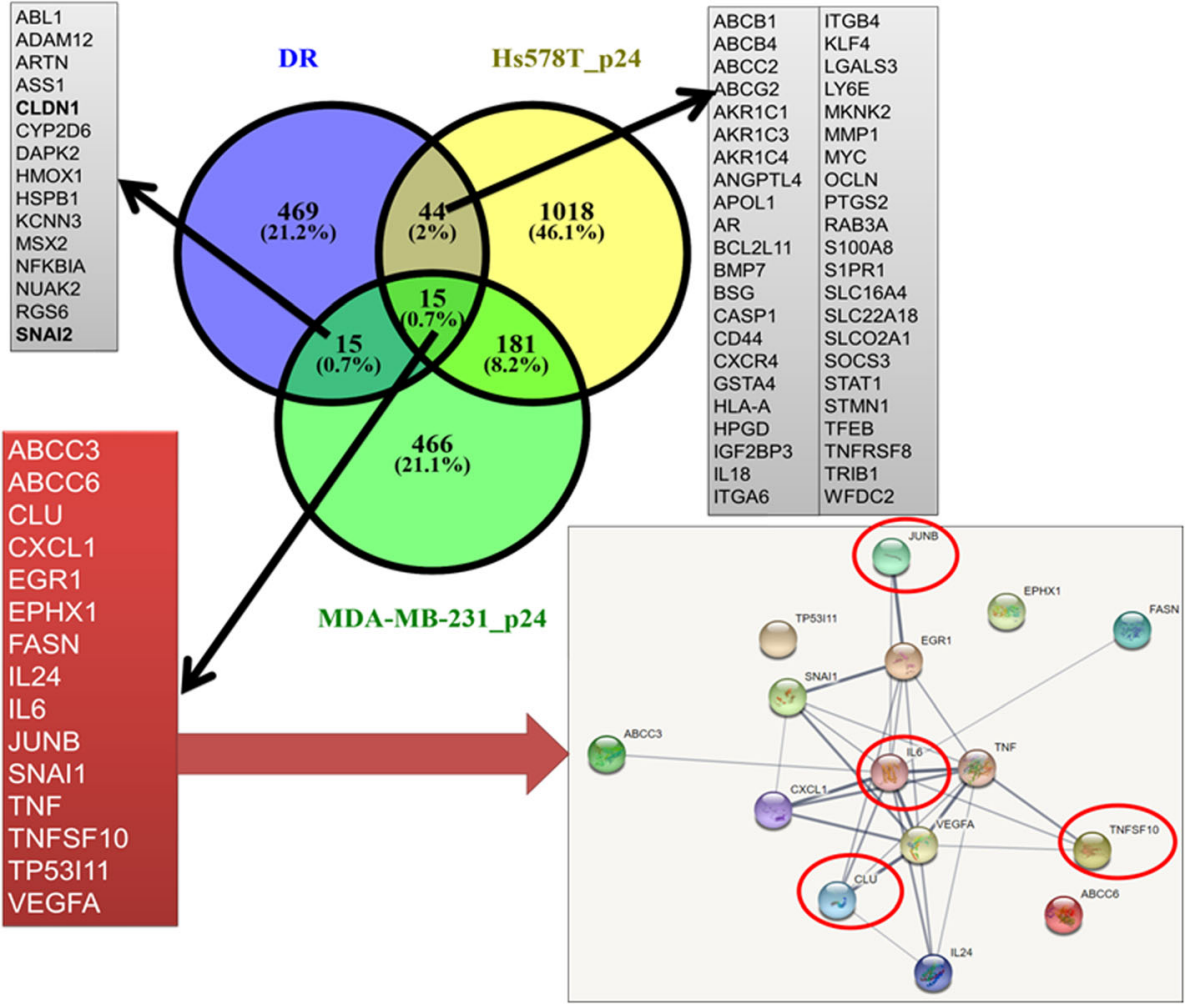
Common drug resistance gene expression signature. **a** Venn diagram used to emphasis the common signature among the drug resistance gene list (downloaded from NCBI) and the overexpressed gene list at P24 on Hs578T and MBA-MB-231 cell lines. **b** Interaction network using String software for common genes signature involved in drug resistance mechanisms

Using Venn diagram, we overlapped and highlighted the common genes between both TNBC cell lines at passage P12 and P24, and drug resistance genes list (list downloaded from NCBI). This overlapping analysis identified 15 genes with a significant involvement in drug resistance mechanisms. Between the 15 genes we identified also *ABCC3* and *ABCC6*, members of the superfamily ABC transporters. Through String software, the interaction network between common genes involved in drug resistance highlighted the *JUNB*, *CLU*, *IL-6*, *TNFSF10* genes as important regulators involved in processes including invasion/metastasis, apoptosis and resistance (Fig. 9).

### Validation of genes involved in drug resistance by RT-PCR technique

**Fig. 10:**
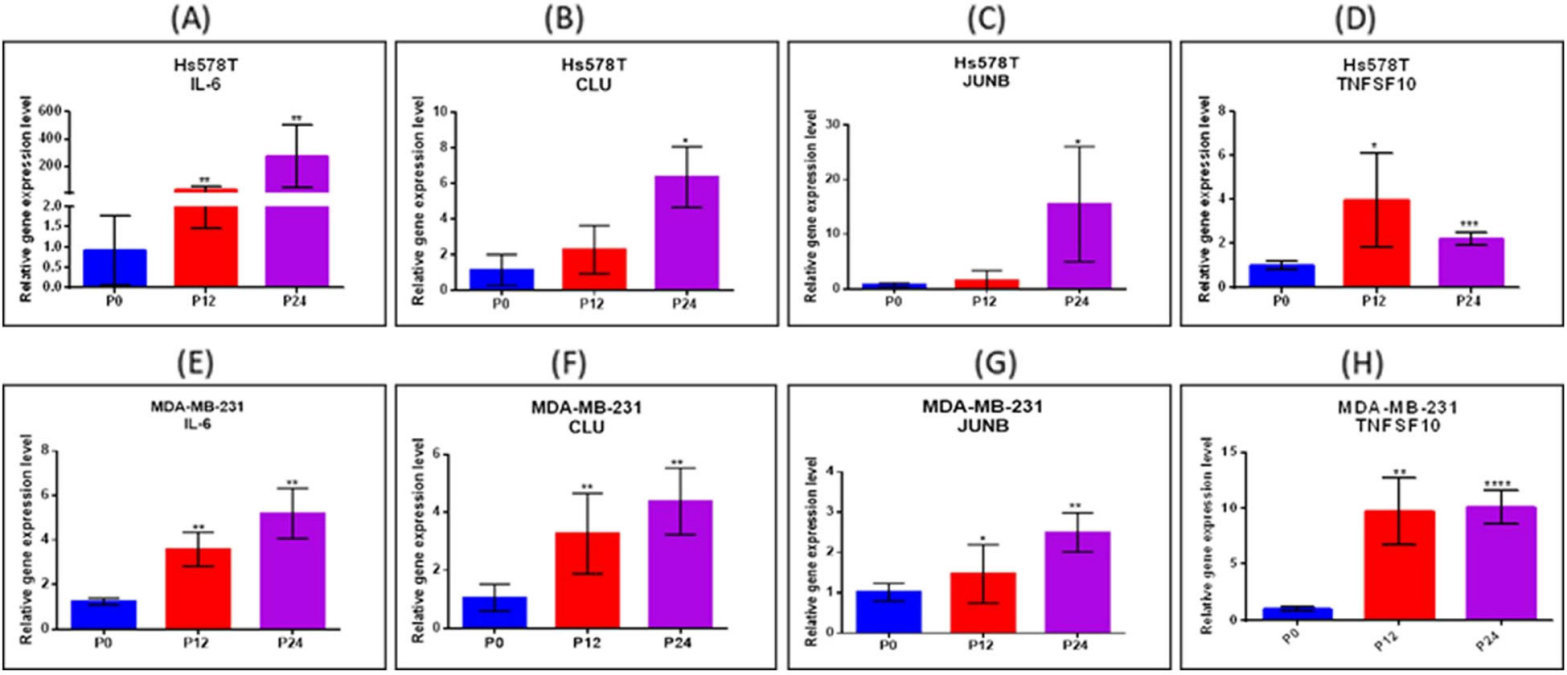
Validation of the effect of doxorubicin therapy using RT-PCR technique on selected genes related to apoptosis processes. Relative gene expression levels are shown for *IL-6*, *CLU*, *JUNB*, *TNFSF10* in treated and untreated group, between P12 and P24 versus P0 on Hs578T and MDA_MB-231 cell lines. The data were normalized to *GAPDH* and *B2M* using ΔΔC_T_ method. Data are presented as mean ± SD using two-sided t-test

The calculation of gene expression FC used the ΔΔC_T_ method and *B2M* and *GAPDH* as the housekeeping gene (Fig. 10). In this section, genes including *IL-6*, *CLU*, *JUNB* and *TNFSF10* were analysed in both TNBC cell lines, specifically for each passage. In Hs578T cell line, the expression levels for *IL-6* (*p* = 0.0382) and *TNSFS10* (*p* = 0.0368) are statistically significant in P12 group compared to P0 group. Regarding the *CLU* gene, the relative expression level is slightly overexpressed, meanwhile *JUNB* gene exhibits no alteration level compared to control group. In the case of P24, the relative expression levels are statistically increased compared to P0 group for *IL-6* (*p* = 0.0453), *CLU* (*p* = 0.0181), *JUNB* (*p* = 0.0083) and *TNFSF10* (*p* = 0.0005). In MDA-MB-231 cell line, we observed that the gene expression profile for the selected genes is statistically overexpressed in P12, respectively P24 group compared to P0 group *IL-6* (*p* = 0.0065, *p* = 0.0020), *CLU* (*p* = 0.0235, *p* = 0.0017), *JUNB* (*p* = 0.0051) and *TNFSF10* (*p* = 0.0012, *p* < 0.0001). Regarding *JUNB* gene, the relative gene expression level is slightly increased but not statistically significant in P12 group compared to control group (P0).

### CXCL1, IL-6 and TNF-α protein quantification using ELISA

**Fig. 11:**
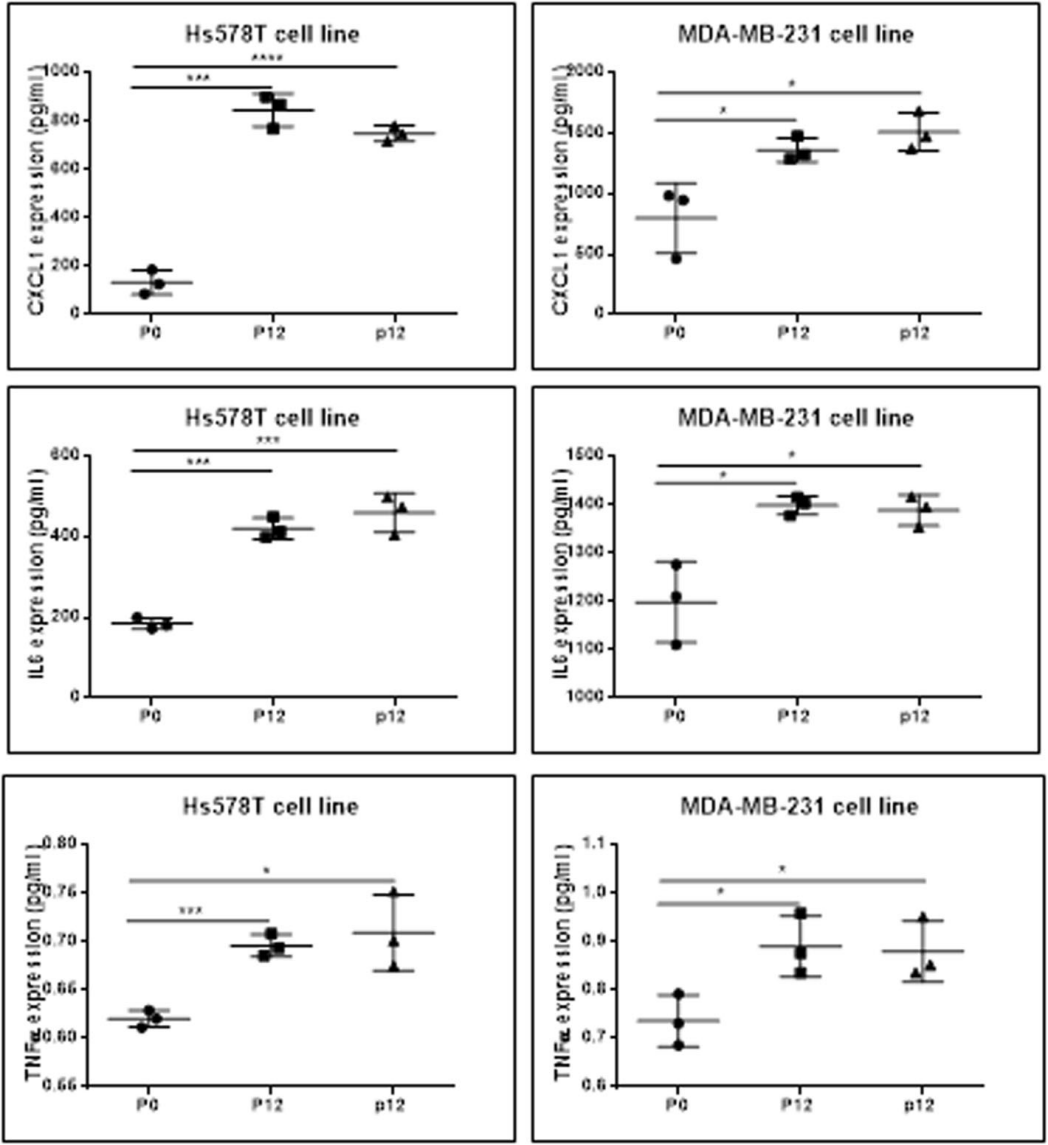
Evaluation of the CXCL1, IL-6 and TNF-α released in cell culture medium by ELISA, for parenteral cells (P0), P12 and P24 for Hs578T and MBA-MB-231 cell lines

As additional validation step was asses the evaluation of CXCL1, IL-6 and TNF-α released for cell culture after 48 h; the results are shown in Fig. 11. Was identified a minimally increased level of CXCL1, IL-6 and TNF-α in the case of P12 and P24 versus parenteral cells, this effect being more intense in Hs578T than in MDA-MB-231 cells.

## Discussions

Doxorubicin remains one of the most active and used chemotherapeutic agent in the treatment of early and advanced breast cancer. Tumor resistance has limited the effectiveness of this therapeutic agent in single drug treatment regiments [15, 21]. It is known that doxorubicin can induce drug resistance, but the most important aspect remains the exact mechanisms behind the resistance that is still poorly understood [22].

As an outcome, some biological processes are modified, including cellular state or adaptive response and chemotherapeutic tolerance, which are reflected through an increase of IC_50_ values at passage P12 and P24 for both cell lines. Also was observed the presence of modifications in the morphological traits and the presence of multinucleated giant cancer cells. This aspect was also found in literature, the frequency of giant cells being correlated with significant anticancer treatment alteration [23]. Other important alteration observed is related to the presence of stress fibers formation (Fig. 1), a mechanism necessary for EMT [24].

NGS is a very valuable tool used in disease characterization, as well as in cancer. Alterations in key genes can affect the response to therapy. In our study, we identified types of mutations involved in several cellular pathways. According to our NGS data, mutations occurred in *TP53* gene were identified. TP53 is a gene that can be used as an independent prognostic factor and associated with a worse prognosis, but further investigation might be needed in order to predict the response to specific therapeutic agents [25]. In Hs578T cell line, *TP53* c.215 C > G mutation is presented only in P12 and P24, meanwhile delGTG *NOTCH1* c.4732_4734, a variant of unknown significance mutation was observed in P24. MDA-MB-231 cell lines have pathogenic mutations in both passages, P12 and P24, A > G *PIK3CA*, drug response mutations C > G *TP53*, as well as variant of unknown significance, G > A *KDR*, c.*37delT *CSF1A*, c.*35instA *CSF1A*, C > T *SMAD4*. These data show the in vitro effect of doxorubicin that can act deeply at the molecular level affecting the structure of DNA toward antitumoral effect.

Further, microarray data for coding and noncoding genes show a differential transcriptomics pattern between both cell lines as well as both passages (P12 and P24). Using bioinformatics tools, we found that most of the enriched GO terms were mainly common among the P12 and P24 for both cell lines treated with doxorubicin. GO analysis revealed that the differentially expressed genes were involved in cellular response, which can be an adaptive response mechanism to doxorubicin, sustained also by the comet assay data.

According to GOrilla software, an activation of the DNA binding activity was observed. This is a mechanism that is still under debate in the case of doxorubicin, but an important aspect is related to the chemoresistance process and correlated with *TP53*-deficient cell context [26]. The DNA damage might induce stabilization of tumor suppressor gene, *TP53*, and might affect cell cycle progression [26] or apoptosis related mechanisms via TNFα signaling (Fig. 7). Moreover, TNF-α was initially found to induce apoptosis in different cancer types by tumor-promoting activities including transformation, proliferation, invasion and metastasis of cancer cells [27]. A high level of TNFα is characteristic to breast cancer and has frequently been associated with a poor prognosis and an aggressive behavior [28]. TNFα has a particular role in enhancing migration and invasion in tumor cells, but the underlying mechanisms are still elusive [29, 30]. In MDA-MB-231 cells was demonstrated that TNF-α increased the expression profile and activity of *MMP-9* by inducing *JUNB* DNA binding activity, thus strengthening the concept of a pro-tumorigenic effect of TNFα in breast cancer [31]. Another process that involves TNF-α activity is the EMT, responsible for the loss of cell adhesion, disruption of cell-cell junctions, extensive actin cytoskeleton reorganization, resistance to cell death, increasing the mobility and invasiveness [32].

Doxorubicin acts as an intercalating agent having the ability to block DNA synthesis and transcription, as well as inhibiting the activity of topoisomerase type II enzyme. This process cause breaks in the genomic DNA that ultimately can lead to apoptosis [33]. These alterations induced in genomic DNA can lead to modifications responsible for the activation of critical biological processes, mainly involved in drug resistance [7]. In Fig. 8 are highlighted the main common genes involved in drug resistance mechanisms. The presence of ATP-binding cassette (ABC) family transports suggest the implication and activation of the mechanisms involved in drug response. *ABCC3* and *ABCC6* members, which participate directly in the active transport of drugs into subcellular organelles or influence drug distribution indirectly, were observed in our study. Balaji et al. showed that overexpression of *ABCC3* is correlated with decreased drug retention; meanwhile knockdown of *ABCC3* increased drug retention and cell death [34]. Overexpression of *ABCC6* is able to confer low levels of resistance to several anticancer agents including doxorubicin, etoposide, daunorubicin and actinomycin [35]. Other gene found in drug resistance network is *JUNB* which has been associated with invasion/metastasis in breast cancer and represents an important target in diseases, associated with EMT. Also, *JUNB* has been involved in the earliest events of resistance development in breast cancer [36]. In addition, *CLU* (Clusterin) is involved in anti-apoptotic processes, development of therapy resistance, induction of EMT, all associated with cancer metastasis. Moreover, *CLU* has the capability to protect metastatic cells from cell death, leading to cell survival in different environment [37]. Also, *CLU* is a stress-activated cytoprotective chaperone targeted and upregulated by a wide range of anticancer therapies that confer treatment resistance [38]. In breast cancer, overexpression of *CLU* was also associated with resistance to neoadjuvant chemotherapy [39].

The significant altered gene expression signature identified can reflect biological regulation and metabolic response in the presence of doxorubicin. We found that TNBC cells developed an adaptive response against the intracellular stress induced by doxorubicin therapy during the stimulation of the resistance process. We identified a 15 gene common signature (*TNF, VEGFA, IL-6, TNFSF10, CLU, ABCC6, EGR1, SNAI1, ABCC3, EPHX1, FASN, CXCL1, IL24, JUNB, TP53I11*) correlated with drug resistance.

## Conclusion

By understanding the molecular bases of chemoresistance in TNBC and functional pathways that are involved in drug resistance mechanisms, we tried to reveal critical aspects to improve prognosis rate and to counteract drug resistance mechanisms. Our study provided important information related to doxorubicin response mechanism correlating data of genetic and transcriptomic alteration with gene networks associated with drug resistance and cellular response. This information could be a valuable starting point for follow-up experiments that might test novel drug targets for anti-cancer treatment in order to prevent activation of drug resistance mechanisms.

## Supplementary Information

**Additional file 1: Figure S1.** The experimental workflow for the multiple dose exposure, at each 4 days cells were passaged and added fresh medium with 50 nm Dox.

## Data Availability

The TCGA material is public available. In the case of plasma microarray data can be added as the row data, or in public repository data.

## Abbreviations

^O^C: Degree Celsius
cDNA: Complementary DNA
CLU: Clusterin
Dox: Doxorubicin
DNA: Deoxyribonucleic acid
DAPI: 4′,6-diamidino-2-phenylindole
EMT: The epithelial–mesenchymal transition
ER: Estrogen
FBS: Fetal bovine serum
HER2: Human epidermal growth factor receptor 2
IC_50_: The half maximal inhibitory concentration
lncRNA: Long non-coding RNAs
μg: Milligram
μl: Milliliter
mM: Millimolar
MTT: 3-(4,5-Dimethylthiazol-2-yl)-2,5-Diphenyltetrazolium Bromide
NGS: Next Generation Sequencing
Ng: Nanogram
nM: Nanomolar
Nm: Nanometer
pM: Picomolar
PR: Progesterone
qRT-PCR: Real-Time Reverse Transcription PCR
RNA: Ribonucleic acid
Rpm: Revolutions per minute
SD: Standard deviation
TNBC: Triple negative breast cancer
TNF-α: Tumor necrosis factor α
