# Correction to: New insights in gene expression alteration as effect of doxorubicin drug resistance in triple negative breast cancer cells

**DOI:** 10.1186/s13046-020-01789-3

**Published:** 2020-12-17

**Authors:** Cristina Alexandra Ciocan-Cartita, Ancuta Jurj, Oana Zanoaga, Roxana Cojocneanu, Laura-Ancuta Pop, Alin Moldovan, Cristian Moldovan, Alina Andreea Zimta, Lajos Raduly, Cecilia Pop-Bica, Mihail Buse, Liviuta Budisan, Piroska Virag, Alexandru Irimie, Sandra Martha Gomes Dias, Ioana Berindan-Neagoe, Cornelia Braicu

**Affiliations:** 1 Research Center for Functional Genomics, Biomedicine and Translational Medicine, “Iuliu Hatieganu” University of Medicine and Pharmacy, Cluj-Napoca, Romania; 2 MedFuture Research Center for Advanced Medicine, “Iuliu Hatieganu” University of Medicine and Pharmacy, Cluj-Napoca, Romania; 3 Laboratory of Radiotherapy, Radiobiology and Tumor Biology, “Prof. Dr. Ion Chiricuta” Oncology Institute, Cluj-Napoca, Romania; 4 Department of Surgical Oncology and Gynecological Oncology, “Iuliu Hatieganu” University of Medicine and Pharmacy, Cluj-Napoca, Romania; 5 Department of Surgery, “Prof. Dr. Ion Chiricuta” Oncology Institute, Cluj-Napoca, Romania; 6 Brazilian Biosciences National Laboratory (LNBio), Brazilian Center for Research in Energy and Materials (CNPEM), Campinas, Sao Paulo, 13083–970 Brazil; 7 Department of Functional Genomics and Experimental Pathology, “Prof. Dr. Ion Chiricuta” Oncology Institute, Cluj-Napoca, Romania

**Correction to: J Exp Clin Cancer Res 39, 241 (2020)**

**https://doi.org/10.1186/s13046-020-01736-2**

Following publication of the original article [1], the authors identified an error in the author name of Sandra Martha Gomes Dias.

The incorrect author name is: Sandra Martha Gomez Diaz

The correct author name is: Sandra Martha Gomes Dias

The author group has been updated above, and the original article has been corrected. No further changes were made to the article.
