# Supplementary figures and images for: New insights in gene expression alteration as effect of doxorubicin drug resistance in triple negative breast cancer cells
Source: J Exp Clin Cancer Res. 2020 Nov 13;39:241. doi: 10.1186/s13046-020-01736-2 (PMC7664031; doi:10.1186/s13046-020-01736-2)

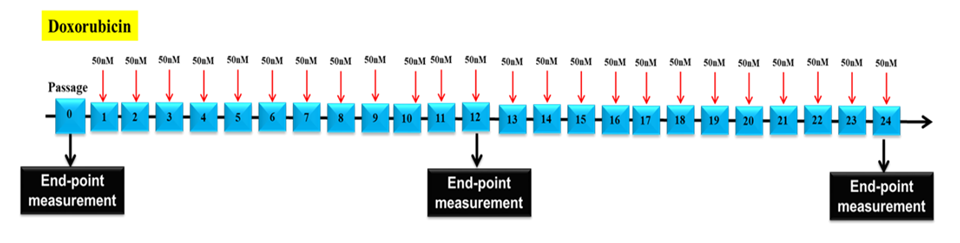

Supplement: Supplementary file 1 — Additional file 1: Figure S1. The experimental workflow for the multiple dose exposure, at each 4 days cells were passaged and added fresh medium with 50 nm Dox. [file 13046_2020_1736_MOESM1_ESM.tif]
